# Response and recovery of Nile tilapia exposed to diesel oil – Behavioral, hemato-biochemical and morphological changes of erythrocytes

**DOI:** 10.1016/j.toxrep.2022.03.039

**Published:** 2022-03-29

**Authors:** AKM Munzurul Hasan, Syed Rubaiyat Ferdous, SM Majharul Islam, Morteza Haghiri, Md Shahjahan

**Affiliations:** a Laboratory of Fish Ecophysiology, Department of Fisheries Management, Bangladesh Agricultural University, Mymensingh 2202, Bangladesh; b Department of Fisheries Biology and Genetics, Bangladesh Agricultural University, Mymensingh 2202, Bangladesh; c School of Science and the Environment, Memorial University-Grenfell Campus, 20 University Drive, Corner Brook, NL, Canada

**Keywords:** Aquatic pollution, Behavior, Blood parameters, Diesel oil, Micronucleus

## Abstract

Pollution caused by petroleum oil in several manners is becoming a threat to aquatic ecosystem. Hence, we carried out an experiment to investigate how diesel oil affects biota behavior, physiological attributes and how they recover by using Nile tilapia (*Oreochromis niloticus*) as a model organism. Nile tilapia of two different treatment groups were exposed to 0.1 mL/L and 0.5 mL/L diesel oil for 7 days. Then both groups were kept in completely diesel oil-free water for 14 days. A control group was maintained throughout the experimental period. We examined the behavioral attributes, hemato-biochemical parameters: hemoglobin (Hb), red blood cell (RBC), white blood cell (WBC) and glucose (Glu), and morphological changes of erythrocytes after diesel exposure and at the end of recovery phase. Our results revealed that there were abnormalities in behavior and significant changes in Hb, RBC, WBC and Glu level in both of the treatment group after 7 days of exposure. Frequencies of erythrocytic cellular abnormalities (ECAs), for example, twin, spindle, elongated, tear drop and erythrocytic nuclear abnormalities (ENAs) like notch nuclei, karyopyknosis, nuclear bud and nuclear bridge were prominent in both groups. However, the amount of anomalies was higher in most if not all the cases in 0.5 mL/L treatment group. Nile tilapia of both groups were quick to recover but the 0.1 mL/L group showed profound recovery than the 0.5 mL/L group. However, in the cases of ECAs and ENAs, recovery of the 0.5 mL/L group was insignificant. Hence, our experimental study concluded that the higher the exposure to diesel oil, higher incidences of major health problems are recorded, seriously piercing the healing system of Nile tilapia.

## Highlights

• Nile tilapia was exposed to 0.1 and 0.5 mL/L diesel oil for 7 days and then diesel oil-free water for 14 days.
• Abnormalities in behavior, blood cells and significant changes in Hb, RBC, WBC, glucose were observed in the exposed tilapia.
• Nile tilapia of both groups were quick to recover and the 0.1 mL/L group showed profound recovery than the 0.5 mL/L group.

## Graphical Abstract

**Figure:**
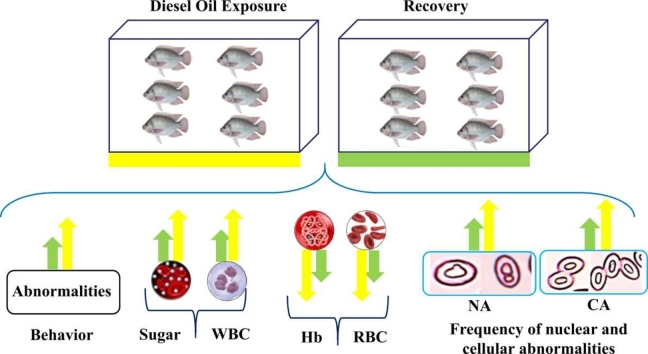

## 1 Introduction

Pollution of aquatic ecosystem has prompted a telltale effect due to the incredible anthropogenic contaminations [1]. Among the several different types of aquatic pollution, petroleum hydrocarbon pollution has become a severe environmental issue due to its composition, with toxic substances, and for its ability to stay in the water for a long time being a stable compound. The fundamental sources of petroleum pollution in aquatic environments are oil transport pipelines, capacity tanks, collision of oil carrying vehicles and the mining of oil shale [2]. Toxic compounds in crude oil lead to acute and chronic toxicity of aquatic animals [3], [4]. It has been reported that crude oil damages the morphology and physiology of fish in different stage and lead to mass mortality [5], [6]. Subsequently, the investigation of oil exposure of fish has gotten importance in the last few decades and researchers have observed the long-term consequences of petroleum hydrocarbons on feeding, growth, behavior, reproduction, and tissue damage of fish [7], [8], [9], [10]. Change in blood parameters can be used for determining the health status of fish [11], [12], [13], [14]. For instance, hemoglobin (Hb) transports oxygen to diverse tissues in most cases, hence the Hb concentration of the blood is frequently utilized to determine anomalies and pathological indicators of fish health [15], [16]. Variation in the white blood cell count and change in blood glucose concentration are employed as biochemical immunosuppressive indicators in fish ([17], [18]). Morphological changes in blood cells are also investigated in order to measure the health status of fish [19], [20]. For example, the formation of micronucleus in erythrocytes can be an indicator of stress induced by pollution in the environment [19], [21], [22], [23].

The Nile tilapia (*Oreochromis niloticus*) is one of the most significant freshwater fish species for global aquaculture, owing to its hardiness and ability to adapt in captivity [24], [25]. The Nile tilapia is a widely used model organism in the field of ecotoxicology [26], [27]**.** Different biochemical reactions have been portrayed in this fish through exposure to lethal compounds [28], [29], [30]. This species is very suitable organism for monitoring the effects of xenobiotics [2]. Several authors have reported the effect of diesel and different lubricant oil on oxidative stress, histopathological alteration of tissues, growth and biotransformation enzymes [2], [26], [31]. Although Nogueira et al. [2] reported the biochemical biomarkers related to oxidative stress in Nile tilapia exposed to diesel oil, no available information regarding the alterations of behavior, hemato-biochemical parameters, erythrocytic abnormalities, and its recovery pattern of Nile tilapia exposed to diesel oil. Therefore, in the present study, we assessed the effect of diesel oil exposure on behavior, hemato-biochemical parameters, erythrocytic abnormalities, and recovery patterns in Nile tilapia.

## 2 Materials and method

### 2.1 Experimental fish

Healthy Nile tilapia fingerlings (mean length; 8.0 ± 0.5 cm and average body weight; 6.0 ± 0.2 g) were collected from Bangladesh Fisheries Research Institute, Mymensingh. The collected fish were acclimatized to the laboratory environment for 15 days in natural photo-regime condition into clean glass aquaria containing 30 L of water. The fish were fed with commercial feed (Mega fish feed, Bangladesh) by 5% of their total body weight twice a day around 9 h and 17 h during the acclimatization period. The experimental procedure and fish used in the experiment have been approved by the Animal Welfare and Ethical Committee, Bangladesh Agricultural University, Mymensingh.

### 2.2 Exposure assessment

The Nile tilapia fingerlings were exposed to three different concentrations (0.0, 0.1 and 0.5 mL/L) of diesel oil for 7 days. The doses were taken on the basis of a study on tilapia by Nogueira et al. [2]. Water was exchanged every 24 h and desired concentrations of diesel oil were added accordingly. At the end of 7 days exposure, eight fish (n = 8) were sacrificed from each concentration.

### 2.3 Recovery assessment

After 7 days of exposure to diesel oil, the rest of fish was reared for next 14 days without diesel oil to monitor the recovery assessment. Water change and feeding remained the same in the exposure and recovery periods. At the end of 14 days post exposure, eight fish (n = 8) were sacrificed from each concentration.

### 2.4 Behavioral analysis

The fish were observed carefully from outside to understand their behavioral pattern in all the aquaria during exposure and post-exposure. Their behavioral activities, such as feeding rate, gulping, gill motion, rate of locomotion etc. were recorded following the previous studies [32]. The findings were presented as severe abnormalities, moderate abnormalities, mild abnormalities, and no abnormalities.

### 2.5 Measurement of hemato-biochemical parameters

On every sampling day, fish were anesthetized through 5 mL/L of clove oil immediately after collected from each aquarium. Blood was sampled from the caudal peduncle of each fish and kept immediately in an autoclaved centrifuge tube with previously added 20 mM EDTA anticoagulant. The blood glucose level (mg/dL) and hemoglobin (Hb) content of fish were measured immediately after the collection of blood from the fish. The glucose and Hb were determined using a blood glucose meter and Hb strip through a digital EasyMate® GHb (Model-ET, 232) monitoring system. Neubauer hemocytometer was used to count the RBC and WBC number under a light microscope described as per Blaxhall and Daisley [33].

### 2.6 Analysis of morphological changes of erythrocytes

A detailed procedure for analyzing of the morphological changes of erythrocytes was described by Shahjahan et al. [19] and Jahan et al. [34]. In brief, blood sample was smeared onto a clean microscopic slide immediately after the blood collection. Blood smear was then fixed by methanol after air-drying for 10 min and finally 5% Giesma solution was used for staining. The slides were kept for air-dried after rinsed with tap water. The slides were subsequently mounted with dibutylphthalate polystyrene xylene (DPX) and preserved in room temperature. An Electronic microscope (MCX100, Micros, Austria) was used to investigate the cellular and nuclear abnormalities of erythrocytes. From each of the slide, at least 2000 cells with intact cellular and nuclear membrane were counted. Erythrocytic cellular abnormalities can be categorized according to their condition. For instance, twin: where two cells are joined by the outer membrane. Spindle shape: the cell have two pointy ends and somewhat rounded in the middle. Elongated cell: the lengths are unusual compared the normal cell. Tear drop: slightly rounded or blunted shape. Different erythrocytic nuclear abnormalities (ENAs) at different sampling point were categorized according to Carrasco et al. [35]. For instance, cells without nuclear material having no nuclear shape were designated as notched nuclei, cells with condensed and clumped chromosomal substances with unusual membranes in the nucleus were considered as karyopyknosis, nuclei having evaginations like a bud were defined as nuclear bud, and thin filaments connecting individual nuclei were classified as nuclear bridge.

### 2.7 Data analysis

Values of all the measured variables were presented as mean ± standard deviation of the mean (SD). One-way analyses of variance (ANOVA) were performed using post hoc Tukey’s test to evaluate the statistically significant difference between the sampling points. To evaluate the significant difference among temperature treatments Mann-Whitney U test and Bonferroni corrections were used. Statistical analyses were performed with PASW statistics 18.0 software (IBM SPSS statistics, Chicago, USA) where p < 0.05 was set as statistical significance value.

## 3 Results

### 3.1 Behavioral changes after exposure to diesel oil

No behavioral alterations were observed in the control group. Gulping and gill motion showed severe abnormalities at a higher concentration (0.5 mL/L) of diesel oil. Moderate abnormalities of locomotion and feeding rate were detected at 0.1 mL/L of diesel oil exposure (Table 1).

**Table 1:** Behavioral changes in Nile tilapia exposed to different concentrations of diesel oil for 7 days and recovery for 14 days.

Parameters	Diesel oil (mL/L)	Exposure (7 days)	Recovery (14 days)
Feeding rate	0.0	−	−
	0.1	++	+
	0.5	++	++
Gulping	0.0	−	−
	0.1	++	+
	0.5	+++	++
Gill motion	0.0	−	−
	0.1	++	−
	0.5	+++	++
Rate of locomotion	0.0	−	−
	0.1	+	+
	0.5	++	+

−, None (0%); +, mild (<10%); ++, moderate (10–50%); +++, severe (>50%)

### 3.2 Recovery of behavioral changes

Feeding rate, gulping and rate of locomotion were showed mild abnormalities during the recovery period exposed to a lower dose of diesel oil. On the other hand, moderate abnormalities of feeding rate and gill motion were found in exposed to a higher concentration of diesel oil (Table 1).

### 3.3 Changes of hemato-biochemical parameters after exposure to diesel oil

**Fig. 1:**
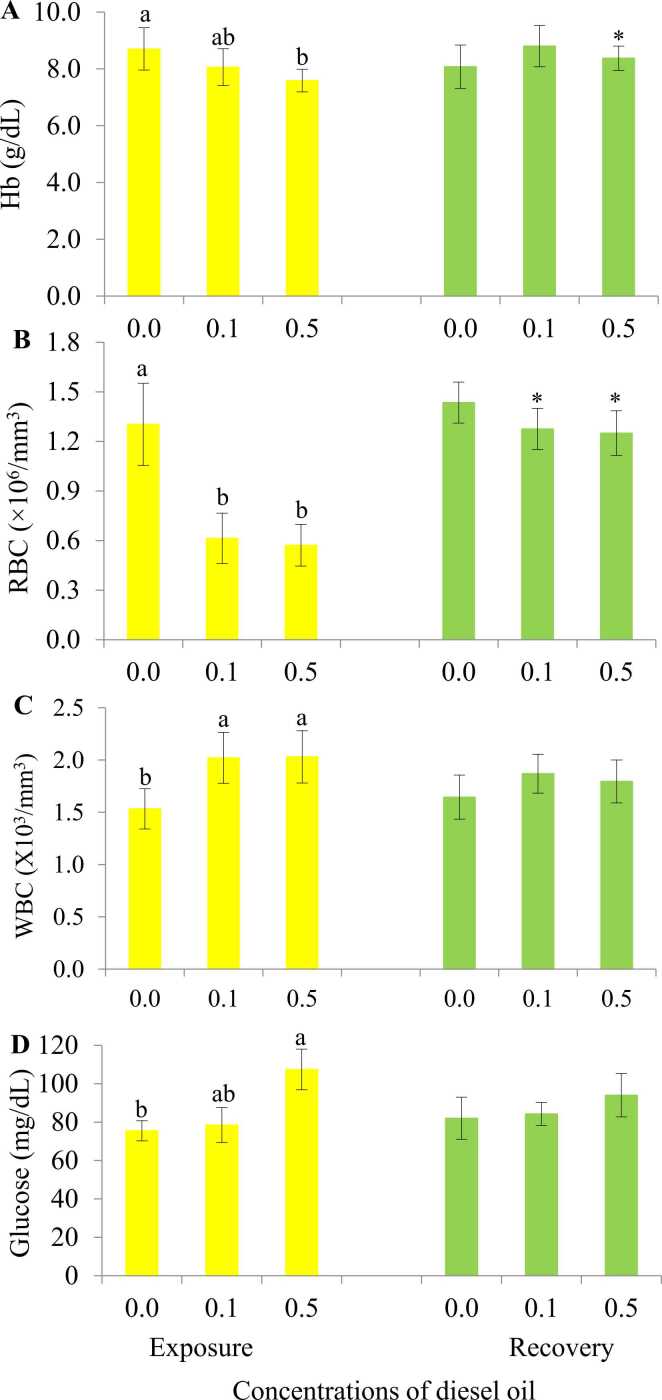
Changes in hemato-biochemical parameters of Nile tilapia exposed to different concentrations of diesel oil for 7 days and recovery for 14 days; a. Hb; b. RBC; c. WBC; and d. glucose. Values with different alphabetical superscripts are significantly (p < 0.05) different. Asterisk (*) indicates the significant difference between exposure and recovery. All values are expressed as mean ± SD.

Level of different hemato-biochemical parameters (Hb, RBCs, WBCs and glucose) of both control and different treatment groups were recorded after 7 day of diesel exposure (Fig. 1). In case of Hb level (g/dL), fish exposed to 0.1 mL/L diesel showed a slight downfall compared to those of the control group while the hemoglobin level of fish exposed to 0.5 mL/L diesel dropped significantly (p < 0.05). RBC numbers (×10^6^/mm^3^) dwindled dramatically (p < 0.05) in both 0.1 mL/L and 0.5 mL/L treatment group as well. On the contrary, the WBC number (×10^6^/mm^3^) displayed an opposite scenario and escalated significantly. When it comes to glucose level (mg/dl), the 0.1 mL/L treatment group showed a small increase whereas fish exposed to 0.5 mL/L diesel exhibited a significant upsurge (p < 0.05).

### 3.4 Recovery of hemato-biochemical parameters

Recovery of Hb, RBCs, WBCs and glucose level were measured after 14 days from day of last diesel exposure (Fig. 1). The control group demonstrated stability in all the different parameters. Hb level (g/dL) went up significantly and showed close resemblance to the control group in both of the treatment groups after 14 days of the recovery period. Same was the case for RBC numbers (×10^6^/mm^3^) in which statistically noteworthy acceleration was noticed (p < 0.01). However, there were no major variations found in WBC numbers (×10^6^/mm^3^) and glucose level (mg/dL) of both of the recovery group.

### 3.5 Aberrations of erythrocytes at different concentrations of diesel oil exposure

**Fig. 2:**
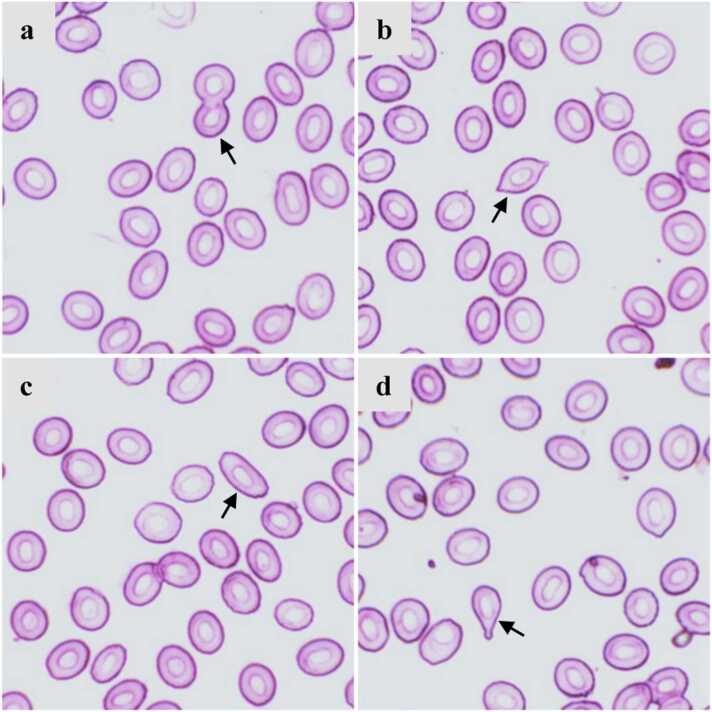
Erythrocytic cellular abnormalities (ECAs) of Nile tilapia exposed to different concentrations of diesel oil for 7 days and recovery for 14 days; a. twin, b. spindle, c. elongated and d. tear drop.

**Fig. 3:**
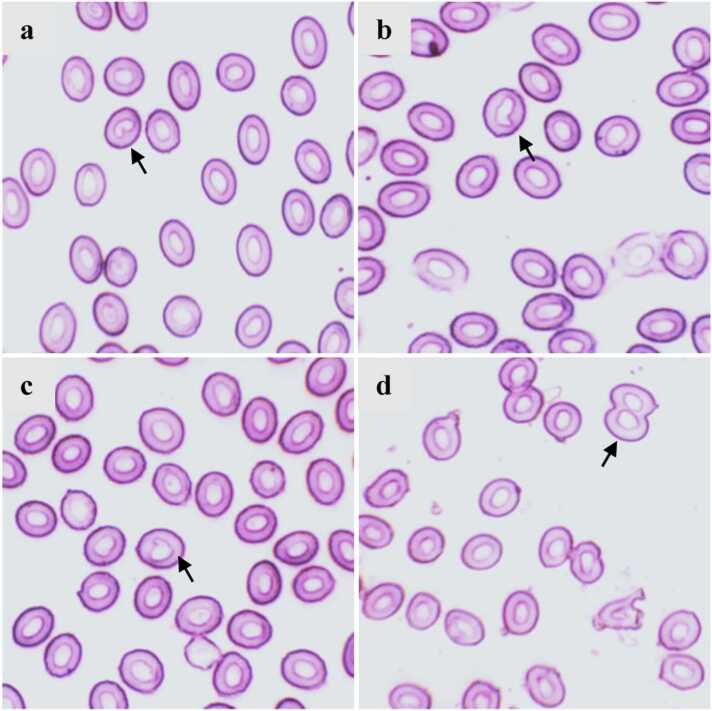
Erythrocytic nuclear abnormalities (ENAs) of Nile tilapia exposed to different concentrations of diesel oil for 7 days and recovery for 14 days; a. notch, b. karyopyknosis, c. nuclear bud and d. nuclear bridge.

Erythrocytic cellular abnormalities (ECAs) were detected in the different group of fish treated with diesel. Twin, spindled, elongated, tear drop (Fig. 2) abnormalities were spotted. Frequencies of ECAs went up significantly (p < 0.05) in both of the treatment groups exposed to diesel in comparison with those of the control group (Table 2). In the similar fashion, erythrocytic neuclear abnormalities (ENAs) were also found significantly higher (p < 0.05) in the two treatment groups (Fig. 3). An ample amount of notch nuclei, micronuclei, nuclei degeneration, nucleus and nuclear bridge were present in both of the treatment group (Table 3). However, though both of the treatment group (0.1 mL/L and 0.5 mL/L diesel exposure) indicated a significant escalation of ECAs and ENAs, the aberrations between the two treatment groups of diesel were hardly noticeable.

**Table 2:** Erythrocytic cellular abnormalities (ECAs) of Nile tilapia exposed to different concentrations of diesel oil for 7 days and recovery for 14 days.

ECA	Diesel oil (mL/L)	Exposure (7 days)	Recovery (14 days)
Twin	0.0	0.47 ± 0.02^a^	0.40 ± 0.08
	0.1	0.53 ± 0.07^a^	0.34 ± 0.18
	0.5	1.04 ± 0.11^b^	0.64 ± 0.13*
Spindle	0.0	0.10 ± 0.13^a^	0.09 ± 0.02^a^
	0.1	0.15 ± 0.18^ab^	0.11 ± 0.05^a^
	0.5	0.23 ± 0.17^b^	0.18 ± 0.09^b^
Elongated	0.0	0.20 ± 0.08^a^	0.18 ± 0.03^a^
	0.1	0.46 ± 0.11^b^	0.24 ± 0.14^ab,^*
	0.5	0.47 ± 0.14^b^	0.42 ± 0.19^b^
Tear-drop	0.0	0.13 ± 0.04^a^	0.14 ± 0.09^a^
	0.1	0.91 ± 0.07^b^	0.27 ± 0.15^ab,^*
	0.5	1.15 ± 0.12^b^	0.91 ± 0.19^b^

Values of a single cellular abnormalities in a column with different alphabetical superscripts are significantly (p < 0.05) different. Asterisk (*) indicates the significant difference between exposure and recovery in the row. All values are expressed as mean ± SD. Three slides were prepared from each fish and 2000 cells were scored from each slide and at least three fishes were analyzed from each group.

**Table 3:** Erythrocytic nuclear abnormalities (ENAs) of Nile tilapia exposed to different concentrations of diesel oil for 7 days and recovery for 14 days.

ENA	Diesel oil (mL/L)	Exposure (7 days)	Recovery (14 days)
Notch nuclei	0.0	0.14 ± 0.03^a^	0.11 ± 0.04^a^
	0.1	0.63 ± 0.09^b^	0.16 ± 0.12^ab,^*
	0.5	0.72 ± 0.14^b^	0.40 ± 0.10^b^
Karyopyknosis	0.0	0.17 ± 0.03^a^	0.14 ± 0.01^a^
	0.1	0.31 ± 0.07^b^	0.18 ± 0.11^a^
	0.5	0.38 ± 0.10^b^	0.22 ± 0.12^a,^*
Nuclear bud	0.0	0.19 ± 0.04^a^	0.12 ± 0.03^a^
	0.1	0.24 ± 0.09^ab^	0.20 ± 0.07^ab^
	0.5	0.33 ± 0.05^b^	0.30 ± 0.06^b^
Nuclear bridge	0.0	0.19 ± 0.09^a^	0.16 ± 0.09^a^
	0.1	0.56 ± 0.13^b^	0.29 ± 0.06^ab,^*
	0.5	0.88 ± 0.17^b^	0.64 ± 0.11^b^

Values of a single cellular abnormalities in a column with different alphabetical superscripts are significantly (p < 0.05) different. Asterisk (*) indicates the significant difference between exposure and recovery in the row. All values are expressed as mean ± SD. Three slides were prepared from each fish and 2000 cells were scored from each slide and at least three fishes were analyzed from each group.

### 3.6 Recovery response of erythrocytes

Fish of the control group displayed substantial constancy in case of ECAs and ENAs throughout the experimental period. Fish exposed to 0.1 mL/L diesel exhibited an instant and decent decrease in ECAs (Table 2) and ENAs (Table 3), whereas fish exposed to 0.5 mL/L diesel were much slower during recovery response.

## 4 Discussion

A petroleum pollutant diesel is considered as a serious threat to water populations due to containing toxic substance and for its ability to stay in the water for a long time being a stable compound. In the present study, several behavioral changes including low feeding rate, gulping, lack of locomotion and abnormalities in the gill motion were observed in both 0.1 mL/L and 0.5 mL/L treatment group and as expected the latter group showed severity in every case because of a higher dose which can be compared with the findings of Armstrong et al. [36] and Bautista et al. [37]. The abnormality in gill motion might have resulted from diesel clogged in the gill. Besides, diesel does not mix with water and made a layer on the water surface which prevented the atmospheric oxygen to mix up and might give rise to lower dissolved oxygen level and gulping. It is possible that it might have happened due to gill injury or change in gill morphology. The osmoregulatory function can be affected by the toxic substances through gill injury and cease the oxygen intake of aquatic organisms [38]. Fish of 0.5 mL/L dose showed inertia and low food intake which is similar to Kori-Siakpere [39] who stated that fish presented to water solvent parts (WSFs) of unrefined petroleum could result in diminished feed admission and low body weight. Dede and Kaglo [40] detailed that the endurance of Nile tilapia diminished by expanding centralization of diesel fuel. After 14 days of the recovery period, the abnormal behavior almost disappeared, especially in the 0.1 mL/L treatment group. Though fish of the 0.5 mL/L treatment group did not show complete normal behavior, the progress was somewhat significant. From this it can be said that if the toxicant level of diesel is low and if the pollutant can be removed from the water fish can recover from their abnormal behavior quickly.

Hematological and biochemical parameters are considered as decent indicators to know whether the fish are in stressful conditions because of aquatic environmental pollutants [13], [41]. We found moderate to severe aberration of hemato-biochemical parameter in our sample treated with diesel and in every case the sample exposed to higher dose (0.5 mL/L) showed greater anomaly compered to lower dose (0.1 mL/L). In our present study, a higher dose of diesel oil exposure in Nile tilapia significantly decreased the Hb and RBC, it is possible that it might have resulted from the breakdown of the hematopoietic system as cessation of the hematopoietic system was severe under the critical condition. The low count of RBC might have resulted from severe anemic state or from hemolysing power of toxicant, particularly on the red cell membrane. Huge decline of Hb level in the 0.5 mL/L treatment group might debilitate oxygen supply to different tissues and might result in a sluggish rate and low energy creation. The critical diminishing in the Hb fixation might have caused due to an expansion in the rate at which the Hb was obliterating or from waning in the pace of Hb synthesis resulting from the toxic effect of diesel. Cessation of Hb and RBCs with an increased percentage of erythroblast abnormalities due to pollution was also found in striped catfish [18] and common carp, *Cyprinus carpio* [15], [42]. Similarly, Gurung et al. [43] reported that crude oil exposure during organogenesis induced greater teratogenic effects on halibut, disturbances cardiovascular flow of embryonic Gulf killifish. We found that both of the treatment group displayed greater WBC number after diesel exposure. The increment in the WBC count can be related to the immune response creation which helps in endurance and recuperation of the fish exposed to diesel [44]. In the current study, the critical expansion in the WBC count may be showed hypersensitivity of leucocytes to diesel and these progressions might be because of immunological responses to create antibodies to cope with the stress initiated by diesel. Changes in blood glucose have been proposed as a helpful general marker of stress in teleost. The acceleration in glucose level also indicated the secretion of cortisol, a stress hormone. Under unpleasant conditions, cortisol provides the body with glucose by taking advantage of protein stores through gluconeogenesis in the liver. This energy can help to battle or escape a stressor. Huge increment of plasma glucose level in the present study might have occurred due to gluconeogenesis to give energy to the expanded metabolic demands as a result of stress. The building and emission of glucocorticoids and catecholamines cause hyperglycemia. Excess stress provokes the production of these hormones from the adrenal tissue, which eventually augments the gluconeogenesis processes in stressed fish [45]. Higher glucose level was also reported as an indicator of stress in several fish species in various stress conditions [46], [47], [48]. So, it is clear that fish can be greatly stressed by diesel, especially when the amount of pollutant is higher. After the recovery phase all the blood parameters were found to be improved and showed similarity with the control group, particularly fish exposed to 0.1 mL/L diesel. Though both of the treatment group recovered greatly from low Hb and RBC level, in case of WBC and glucose level the improvement were less significant which indicate that the immune system of fish is still in action and fish have not completely recovered from stress.

We found several abnormalities in the erythrocytes of the treatment group. The frequencies of aberrations were higher in the 0.5 mL/L treatment group compared to the 0.1 mL/L treatment group. As erythrocytes react to environmental stressors, any alteration (cellular and nuclear) can be read as the presence of toxicants in the water. Erythrocytic cellular abnormalities for example, twin, elongated, spindle shaped, tear drop shaped were observed. It could be resulted from the increase in lipid peroxidation in erythrocytes induced by stress due to diesel exposure [21]. Toxic compound in diesel can disrupt the chain of cellular modification and may lead to hypoxic condition. As a result it can desolate the ATP which may disturb the structure of erythrocyte. Abnormities in nucleus for instance, nuclear bridge, micronucleus, notched nuclei were observed in the treatment group which indicate the genotoxicity of diesel oil [49], [50]. Previous report shows that, toxic compound can potentially disturb the structure of cell membrane, metabolism and ion permeability of erythrocyte which can damage the erythrocyte formation morphologically. Crude oil causes micronuclei development and other nuclear abnormalities in the erythrocytes and cephalic kidney of Atlantic cod, *Gadus morua* and turbot, *Scophthalmus maximus* [51]. After 14 days of the recovery period the frequencies of ECAs and ENAs started to vanish and the improvement was much higher in the 0.1 mL/L treatment group. However, the other group (0.5 mL/L) did not show any significant improvement. Though we found the blood parameters like Hb, RBC, WBC, glucose level improved in quite a good way in this group, but it was not the case when it comes to erythrocytic abnormalities. It was beyond were capacity to determine whether the damages were permanent or would it lead to a severe health problem of fishes in the 0.5 mL/L treatment group.

## 5 Conclusions

The present experimental study reveals that diesel have severe effect on the behavior, hemato-biochemical parameters and morphology of erythrocytes of a fish and can lead to extreme metabolic stress even if the exposure is for a short time and in a lower amount. It also showed that fish can quickly recover from the abnormalities given that the pollutant’s intensity is lesser and completely absent while recovering. But if the concentration is higher, the consequences may be harsh and recover is not completely certain, particularly, in the cellular level. More research warrants knowing whether the toxicants in diesel concentrate in the cellular system and at what extent it can damage different organs when the exposure is greater.

## Ethics approval

The total procedure conducted in this experiment was approved by the Animal Care and Use committee of Bangladesh Agricultural University, Mymensingh (Approval Number: BAU-FoF/2020/004).

## CRediT authorship contribution statement

AKM Munzurul Hasan and Syed Rubaiyat Ferdous performed the experiment, collected data and drafted the manuscript. SM Majharul Islam participated in data collection. Morteza Haghiri edited the manuscript. Md Shahjahan assisted in the experimental design and edited the manuscript. All authors reviewed and approved the final manuscript.

## Declaration of Competing Interest

None.

## Data Availability

The data that support the outcomes of this study are available on request from the corresponding author [M Shahjahan].
